# Photoregulation in a Kleptochloroplastidic Dinoflagellate, *Dinophysis acuta*

**DOI:** 10.3389/fmicb.2016.00785

**Published:** 2016-05-30

**Authors:** Per J. Hansen, Karin Ojamäe, Terje Berge, Erik C. L. Trampe, Lasse T. Nielsen, Inga Lips, Michael Kühl

**Affiliations:** ^1^Marine Biological Section, Department of Biology, University of CopenhagenHelsingør, Denmark; ^2^Marine Systems Institute, Tallinn University of TechnologyTallinn, Estonia; ^3^Centre for Ocean Life, DTU Aqua National Institute for Aquatic Resources, Technical University of DenmarkCharlottenlund, Denmark; ^4^Plant Functional Biology and Climate Change Cluster, University of Technology SydneySydney, NSW, Australia

**Keywords:** acquired phototrophy, *Dinophysis*, kleptochloroplasts, photoregulation, photosynthesis

## Abstract

Some phagotrophic organisms can retain chloroplasts of their photosynthetic prey as so-called kleptochloroplasts and maintain their function for shorter or longer periods of time. Here we show for the first time that the dinoflagellate *Dinophysis acuta* takes control over “third-hand” chloroplasts obtained from its ciliate prey *Mesodinium* spp. that originally ingested the cryptophyte chloroplasts. With its kleptochloroplasts, *D. acuta* can synthesize photosynthetic as well as photoprotective pigments under long-term starvation in the light. Variable chlorophyll fluorescence measurements showed that the kleptochloroplasts were fully functional during 1 month of prey starvation, while the chlorophyll *a*-specific inorganic carbon uptake decreased within days of prey starvation under an irradiance of 100 μmol photons m^-2^ s^-1^. While *D. acuta* cells can regulate their pigmentation and function of kleptochloroplasts they apparently lose the ability to maintain high inorganic carbon fixation rates.

## Introduction

Some free-living phagotrophic protists sequester chloroplasts from their algal prey and utilize them for shorter or longer time; a life style that is common among ciliates, dinoflagellates and radiolarians (e.g., Stoecker et al., 2009; Johnson, 2011). In many such species, however, other prey cell organelles are retained as well. Red tide ciliates, *Mesodinium* spp., ingest certain cryptophyte prey species and sequester not only the chloroplasts (Johnson et al., 2006; Moeller et al., 2011; Hansen et al., 2012), but also a number of other prey cell organelles, such as mitochondria, the prey nucleus, and the nucleomorph (a reduced former nucleus of an earlier endosymbiont found in cryptophytes). These red-pigmented *Mesodinium* spp. can keep the ingested prey organelles functionally active for several months gaining enough photosynthate for survival in periods of low prey abundance (Johnson and Stoecker, 2005; Hansen and Fenchel, 2006; Johnson et al., 2006, 2007; Smith and Hansen, 2007). In fact, the ingestion of only a single cryptophyte prey cell per day (∼1% of daily carbon needs) is sufficient to maintain maximum growth rate (Smith and Hansen, 2007). The red-pigmented *Mesodinium* spp. can control the division of its ingested prey organelles (Hansen and Fenchel, 2006), but this ability seems to get lost after a few cell divisions in prey-starved cultures probably due to loss of prey nuclei material (Johnson et al., 2007; Moeller et al., 2011). Red-pigmented *Mesodinium* spp. display photoacclimation, i.e., increases in cellular photosynthetic pigments at low irradiance and a change in photosynthesis vs. irradiance response curves (Johnson et al., 2006; Moeller et al., 2011).

In other protist species, only prey chloroplasts are sequestered and typically only remain functional in the predator cell for hours up to a few days. However, in some cases such kleptochloroplasts are kept active over several weeks, allowing kleptochloroplastidic predators to survive periods of prey starvation a lot better than purely heterotrophic protists (Stoecker et al., 2009). One conspicuous case is found among the dinoflagellate genus *Dinophysis*, which are associated with diarrheic shellfish poisoning worldwide. Species within this genus have chloroplasts of cryptophyte origin and the first culture of a *Dinophysis* spp. was established by feeding it a red-pigmented *Mesodinium* spp. suggesting that its cryptophyte chloroplasts were supplied from the ciliate prey (Park et al., 2006). This was later confirmed by Kim et al. (2012) and *Dinophysis* spp. can thus employ “third-hand” chloroplasts. A total of eight species have now been cultured all relying on red-pigmented *Mesodinium* spp. as prey (Hansen et al., 2013). *Dinophysis* spp. use a peduncle to suck out the contents of the ciliate prey. *Dinophysis* spp. do not ingest cryptophytes directly and thus rely entirely on *Mesodinium* spp. for the supply of chloroplasts and food. There are no direct evidence suggesting that *Dinophysis* spp. may feed other protists, detritus, or bacteria (Poulsen et al., 2011; Hansen et al., 2013).

It has recently been shown that *Dinophysis acuta* and *D. caudata*, grown together with red *Mesodinium* spp. under low irradiance have an elevated cellular Chl *a* content compared to cells grown at high irradiance (Rial et al., 2013). *Dinophysis* cells grown under low irradiance contain more and larger kleptochloroplasts as compared to cells grown at high irradiance (Nielsen et al., 2012, 2013). This suggests that *Dinophysis* spp. may have the capacity to photoregulate, but how is this possible when neither prey nuclei nor nucleomorphs are retained by *Dinophysis* spp. remains unknown.

The apparent regulation of kleptochloroplasts in *Dinophysis* spp. could work in three ways, alone or in combination: (i) Behavioral regulation: *Dinophysis* spp. may increase the number of chloroplasts incorporated via ingestion when grown under low irradiance and may fuse ingested chloroplasts leading to the observed increase in chloroplast size and cellular Chl *a* content; (ii) Photoregulation: *Dinophysis* spp. may be able to produce Chl *a* and other photosynthetic pigments, while the photosynthetic light response curves are unaffected; (iii) Photoacclimation: If *Dinophysis* spp. perform photoacclimation, an increase in cellular Chl *a* concentration as well as a change in the photosynthetic light response curve is expected. This would indicate full control over the acquired chloroplasts. In the latter two cases, the genes involved in either of such regulations must have been transferred to the host cell. A few chloroplast housekeeping genes have been found in the genome of *Dinophysis acuminata*, but it is unlikely that they alone allow for either photoregulation or photoacclimation (Wisecaver and Hackett, 2010).

In the present study, we investigated the photoregulation potential of *D. acuta* cells deprived of prey while being subjected to low irradiance to test whether photoregulation or photoacclimation occurs in sequestered chloroplasts of cryptophyte origin. For this, we employed a suite of different experimental techniques quantifying cell devisions, photosynthetic performance, inorganic carbon (*C*_i_) uptake, and respiration of *D. acuta* in cultures starved at the initiation of the experiments and incubated at different irradiance levels. We hypothesize that *D. acuta* is only capable of the first of the three options listed above, behavioral regulation, and thus will display no, or limited, control over its kleptochloroplasts during starvation. This would include the lack of ability to synthesize Chl *a* and limited adaptation of photosynthetic light curve response parameters. Thus, we expect to find an exponential decline in cellular Chl *a* content as well as declining values of *C*_i_ assimilation rate and photosynthetic performance, as cells divide during prey starvation.

## Materials and Methods

### Organisms and Culture Conditions

Cultures of the cryptophyte *Teleaulax amphioxeia* (K-1837; SCCAP) and the ciliate *Mesodinium rubrum* (MBL-DK2009) were established from water samples from Helsingør Harbor in 2009. Cultures of *M. rubrum* were fed *T. amphioxeia* at a predator:prey ratio of 1:10 twice a week. Cultures of *D. acuta* were established in June 2010 from the North Sea (Nielsen et al., 2013). *M. rubrum* was added as prey organism twice per week at a predator:prey ratio of ∼1:10 to allow mixotrophic growth. Only *M. rubrum* cultures that had completely removed their cryptophyte prey were used to feed *D. acuta*. All three species were grown in f/2 medium (Guillard, 1983) based on autoclaved seawater with a salinity of 32, a dissolved inorganic carbon (DIC) concentration of 2.3 ± 0.1 mmol l^-1^ and a pH of 8.0 ± 0.05. pH was monitored directly in the flasks with a SenTix41^®^ pH electrode (WTW, Germany) connected to a pH meter (WTW, pH 3210, Germany), and calibrated by measurements in pH 7 and pH 10 standard buffers (WTW, Technischer, NIST, buffers). All organisms were grown at 15.0 ± 1.0°C in a temperature regulated room, under a photon irradiance of 100 μmol photons m^-2^ s^-1^ (PAR, 400–700 nm), as controlled by a timer to a light:dark cycle of 16:8 h, unless otherwise stated. Culture flasks were placed on a glass table with light coming from below. Light was provided by cool white fluorescent tubes (OSRAM 58W, 840) and photon irradiance was measured (in air) at the level of incubation flasks using a light meter equipped with a spherical quantum sensor (ULM & US-SQS/L, Walz GmbH, Germany). All cultures were xenic.

### Experimental Design

#### Experiment 1

This experiment was designed to study photoregulation in a recently prey starved culture of *D. acuta*. A culture of *D. acuta* was maintained in 750 ml tissue culture flasks filled with 500 ml culture medium under a photon irradiance of 100 μmol photons m^-2^ s^-1^ with prey for at least 2 weeks prior to the experiment and was eventually allowed to deplete its ciliate prey (below detection limit which was a few cells ml^-1^). At the initiation of the experiment, subsamples of the culture were split in two and poured into 270 ml tissue culture flasks to capacity at a *D. acuta* cell density of 200 cells ml^-1^ (in triplicate). One set of flasks were maintained at 100 μmol photons m^-2^ s^-1^ (*I*_100_), while the other set of flasks was shifted to a photon irradiance of 15 μmol photons m^-2^ s^-1^ (*I*_15_). Subsamples were withdrawn on day 3, 6, 9, 13, 16, and 29 (*I*_100_), and day 4, 7, 10, 14, 17, and 30 (*I*_15_) for measurements of cell concentration, ^14^C fixation, algal pigment concentration using both fluorometry and HPLC techniques, photosynthetic capacity using variable chlorophyll fluorimetry, and O_2_ optode-based respiration measurements (see descriptions of these techniques below).

pH was checked on each sampling occasion to avoid physiological effects of elevated pH in laboratory cultures (Hansen, 2002). Any cryptophytes left in the incubation flasks at the start of the experiment grew to concentrations where they could affect pH, especially at *I*_100_. As the *Dinophysis* culture increased in cell concentration they also affected the pH of the culture medium. Therefore, we removed most of the culture medium once each week during the experiment, using gentle inverse filtration, and applied fresh f/2 culture medium to ensure optimal growth conditions (i.e., nutrients, pH, etc.). A plastic tube with plankton gauze (mesh size 20 μm) attached to the tip was lowered directly into the experimental flasks and ∼90% “old” growth medium was removed gently using a pipette and replaced with fresh growth medium. This was repeated three times each time.

#### Experiment 2

This experiment was carried out to measure Chl *a*, and physiological rates in well fed cultures of *D. acuta* at an irradiance of 100 μmol photons m^-2^ s^-1^. Cultures were maintained in 750 ml tissue culture flasks filled with 500 ml culture medium prior to the experiment. At the initiation of the experiment, 270 ml tissue culture flasks were filled to capacity with an initial concentration of 400 *D. acuta* cells ml^-1^ and 1000 *M. rubrum* cells ml^-1^ (in triplicate). Samples were withdrawn three times over 6 days for measurements of cell concentration, ^14^C fixation, photosynthetic capacity using variable chlorophyll fluorimetry, and O_2_ optode-based respiration measurements (see descriptions of these techniques below). Fresh growth medium was added at each sampling to replace the water volume removed. *Dinophysis* cells were picked individually using a drawn Pasteur glass pipette and transferred to clean growth medium twice to remove all *M. rubrum* cells before measurements.

### Cell Enumeration

Cells were fixed in Lugol’s solution (1% final conc.) and were enumerated using a Sedgewick Rafter cell and an inverted microscope (Olympus^®^, CK-40). A minimum of 200 *Dinophysis* cells were counted each time and all samples were checked for possible lefteover of ciliate prey cells.

### Photosynthetic Pigments

#### Fluorimetric Measurements of Algal Pigments

Cellular chlorophyll concentration was measured by extracting chlorophyll of single cells according to Skovgaard et al. (2000). One ml of 96% ethanol was added to the borosilicate measuring vials. A total of 80 individual *Dinophysis* cells were pipetted directly into the ethanol and placed in the dark. This process took place in a temperature-regulated room kept at experimental temperature. The chlorophyll *a* concentration (μg Chl *a* l^-1^) was measured in the extract after 20 min using a bench-top fluorometer (Trilogy, Turner designs, USA) equipped with the manufacture’s module for Chl *a* determination. The fluorometer was calibrated against a pure Chl *a* standard (2.13 mg Chl *a* l^-1^) of cyanobacterial origin (DHI, Hørsholm, Denmark).

Additionally, we measured the *in vivo* chlorophyll, and phycoerythrin fluorescence using the appropriate filter set modules for the fluorimeter. For this, 80 individual *Dinophysis* cells were isolated and transferred into plastic measuring cuvettes containing 1 ml of fresh medium. The cells were kept in suspension by carefully blowing air with a pipette immediately before the measurements. Due to high variation between single measurements, the *in vivo* fluorescence was measured every second and averaged over a period of 10 s.

#### Photopigment Analysis

We analyzed the pigment composition of *D. acuta* with high-pressure liquid chromatography (HPLC) using a slightly modified method from Frigaard et al. (1996) measuring the major light harvesting pigment Chl *a*, the photoprotective pigment alloxanthin, and the ratio between the two in *D. acuta* throughout the starvation experiments. For pigment conservation prior to HPLC analysis, 200 *D. acuta* cells were picked with a micropipette and transferred twice into fresh growth medium, before they were spun down at 10 g for 5 min. The supernatant was removed, and the pellet was frozen at –80°C. Before HPLC analysis, each pellet containing 200 *D. acuta* cells was resuspended for pigment extraction in 125 μl of an acetone-methanol (7:2, vol/vol) mixture and sonicated using an ultrasonicator (Misonix 4000, Qsonica LLC., Newtown, CT, USA) under dim light for 20 s. The sonicated cells were extracted in darkness on ice for 2 min. The extracts were briefly centrifuged to pellet cell debris, and the supernatants were mixed with 15 μL ammoniumacetate (1 M) in 0.3 ml HPLC vials. A 100 μL sample of the mixture was then immediately injected into the HPLC.

Pigment extracts were separated and analyzed by a diode array detector connected to the HPLC system (HPLC-DAD & Agilent 1260 Infinity, Agilent Technologies, Santa Clara, CA, USA) fitted with a Nova-pak C18 column (dimensions: 3.9 × 300 mm), detecting specific absorption wavelengths of compounds. The extracts were run at a column temperature of 30°C for 69 min. Injected extracts moved with a flow-rate of 1.0 ml min^-1^ in solvent A (methanol:acetonitrile:water, 42:33:25, vol/vol/vol), and solvent B (methanol:acetonitrile:ethyl acetate, 50:20:30, vol/vol/vol). In the separation process, the mobile phase changed linearly from 30% solvent B at the time of injection to 100% at 52 min, staying at 100% for 15 min and then falling back to 30% within 2 min. Chl *a* and alloxanthin were identified manually from HPLC chromatograms, and pigment ratios were calculated from the derived integrated peak areas using the manufacturers software (OpenLAB CDS ChemStation Edition, Agilent Technologies, Santa Clara, CA, USA).

#### Variable Chlorophyll Fluorescence Analysis

We assessed the photosynthetic capacity of *D. acuta*, mainly by means of evaluating the dark adapted maximum PSII quantum yield, *F*_v_/*F*_m_ as measured by the pulse saturation method with pulse-amplitude modulated (PAM) fluorimeters (Schreiber et al., 1994). Individual cells were imaged with a variable chlorophyll fluorescence imaging system (RGB Microscopy PAM, Walz GmbH, Germany; Trampe et al., 2011), while bulk culture samples were investigated in a cuvette-based chlorophyll fluorimeter (MULTI-COLOR-PAM; Walz GmbH, Germany). Detailed descriptions of the two types of PAM measurements, and definitions of the parameters used, are provided in the Supplementary Material, Section Material and Methods.

##### Sample preparation – Imaging PAM

1.5 ml subsamples of *D. acuta* culture were transferred to 1.5 ml tubes and centrifuged at 43 g for 2 min. The 1.4 ml supernatant was removed, and the pellet was resuspended in the remaining 0.1 ml yielding a concentrated sample facilitating a low search time for cells of interest at high magnification under the microscope. Control experiments showed no significant effect on cell structure or photosynthetic capacity by the treatment (data not shown). A droplet (16.5 μL) of concentrated sample was transferred to a special treated microscope slide displaying a permanently positive charge (Superfrost Ultra Plus, Thermo Scientific, Gerhard Menzel GmbH, Braunschweig, Germany) resulting in good cell adhesion by electrostatic attraction, limiting motion of cells during imaging. Variable chlorophyll fluorescence imaging measurements rely on recording of consecutive images when calculating photosynthetic parameters, and it is thus crucial that there is no cell movement during the saturation pulse. The sample was sealed with a cover glass using petroleum jelly (Vaseline^TM^) at the periphery to avoid evaporation, and the slide was placed at 12°C in a thermostated slide holder (see details in Trampe et al., 2011).

##### Sample prepation – MULTI-COLOR PAM

For each sample, 1000 (Experiment 1) or 300 (Experiment 2) cells were isolated with a drawn out Pasteur pipette and transferred several times through sterile-filtered seawater before final transfer into quartz cuvettes and the volume was adjusted to 1 ml using 30 psu sterile-filtered f/2 medium. Samples were stirred with a magnetic stirrer during measurements.

### Inorganic Carbon Uptake and Respiration

#### ^14^C Incorporation

Subsamples (1–2 ml) were removed from each culture flask for photosynthesis measurements. A total of 80 *D. acuta* cells were picked from the subsamples and washed in f/2 medium by micromanipulation using a stereoscope to remove all prey. A total of 40 *D. acuta* cells were transferred to each of two 20 ml glass scintillation vials containing 2 ml f/2 medium, and 20 μl of a NaH^14^CO_3_ stock solution (specific activity = 100 μCi ml^-1^; Carbon-14 Centralen, Denmark) was added. One vial was incubated for ∼3 h under the treatment irradiance, while the other was kept in complete darkness (by wrapping in several layers of aluminum foil). After incubation, a 100 μL sub-sample was transferred to a new vial containing 200 μl phenylethylamine for determination of specific activity (see Skovgaard et al., 2000 for a detailed method description). The remaining 1.9 ml were acidified with 2 ml 10% glacial acetic acid in methanol, and evaporated overnight at 60°C to remove all inorganic carbon. The residue was then re-dissolved in 2 ml Milli-Q water. All vials were treated with 10 ml Packard Insta-Gel Plus scintillation cocktail, and disintegrations per minute were measured using a Packard 1500 Tri-Carb liquid scintillation analyzer with automatic quench correction. The photosynthetic activity (PA, pg C cell^-1^ h^-1^) per cell was calculated as follows:

PA =DPM×[DIC]C14×h×N

where DPM is disintegrations min^-1^ (in 1.9ml) in the light corrected for dark values, DIC is the concentration of DIC (pg C ml^-1^), ^14^*C* is the specific activity (disintegrations min^-1^ ml^-1^), *h* is the incubation time, and *N* is the number of cells in the vial (1.9 ml). DIC concentrations were measured on 1 ml subsamples using an infrared gas analyzer (ADC 225 Mk3 Gas analyzer, Analytic Development Co. Ltd., Hoddesdon, England) as described in detail elsewhere (Nielsen et al., 2007). Glass vials with screw caps were used for DIC samples allowing no headspace, and the samples were analyzed within a few hours.

#### Respiration Measurements

Respiration rates of *D. acuta* were measured in 1.8 ml glass vials (*n* = 4) equipped with calibrated optical O_2_ sensor spots with optical isolation (Pyroscience GmbH, Germany). The O_2_-dependent luminescence of each sensor spot was monitored contactless across the vial wall using an optical fiber cable fixed at one end to the glass vial using a solid plexi-glass adaptor and connected at the other end to a fiber-optic O_2_ meter (FireSting, PyroScience, Germany). The sensor spot readout was calibrated from readings in anoxic and fully aerated medium. A total of 400 *Dinophysis* cells were isolated by micropipetting and washed 3 times in sterile filtered (pore size 0.22 μm) f/2 culture medium prior to addition to each of the 4 glass vials containing 1 ml sterile f/2 medium. A glass bead was added to each of the vials that were filled to capacity with sterile filtered medium and carefully sealed avoiding any air bubbles inside the closed vials. The vials were mounted on a modified whirley mixer run at low speed to homogenize internal O_2_ gradients in the sample (Trampe et al., 2015). The measurements were done in darkness in a temperature regulated room (15°C), and O_2_ was measured with the built-in temperature correction of the O_2_ meter. The O_2_ concentration in the glass vials was measured for 1–3 h, and the linear decrease in O_2_-concentration (μmol l^-1^ s^-1^) was converted into cellular respiration rate (pg C cell^-1^ day^-1^) by assuming a respiratory quotient of 1.

## Results

### Changes in Photosynthetic Pigments in *D. acuta* Starved of Prey

**FIGURE 1:**
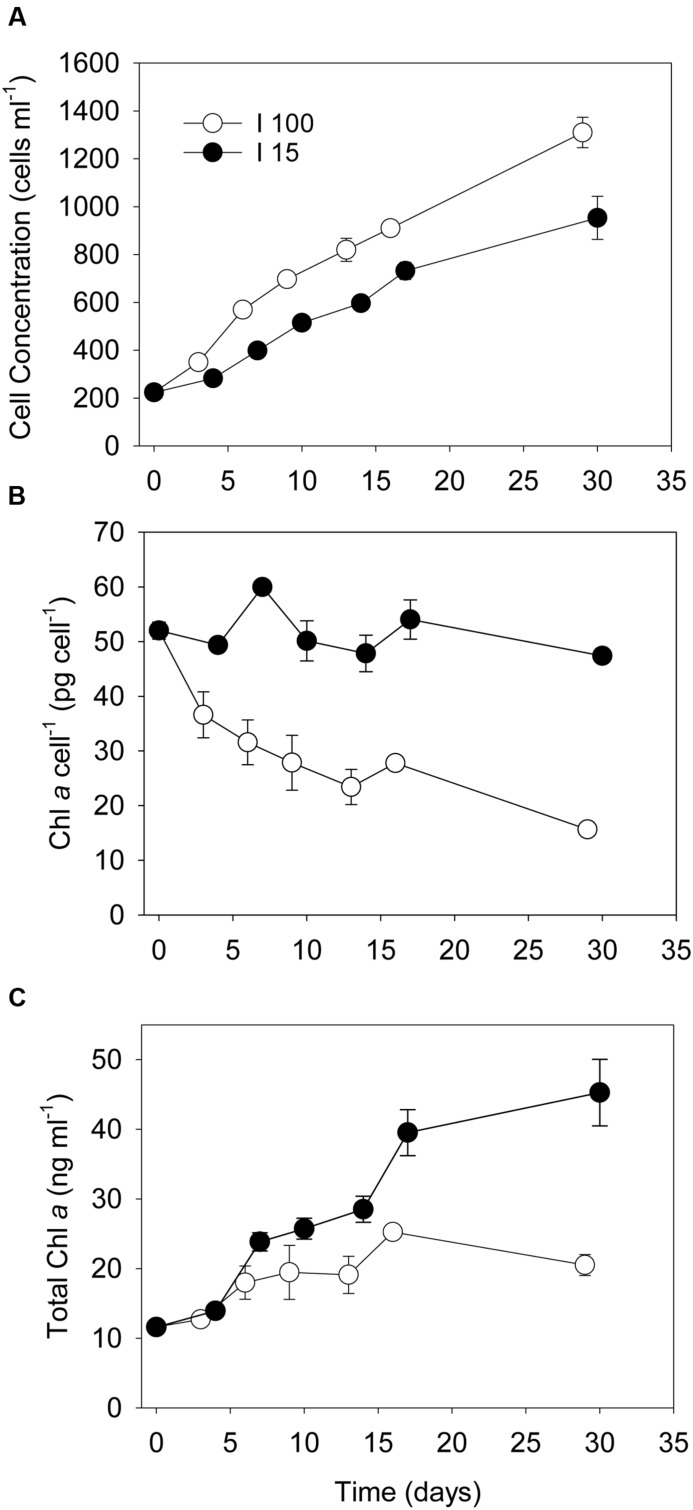
**Developments in *Dinophysis acuta* cell concentrations, cellular Chl *a* content and total Chl *a* concentrations when subjected to prey starvation at the start of the experiment and incubated for 1 month under an irradiance of **(o)** 100 μmol photons m^-2^ s^-1^ (*I*_100_) and **(●)** 15 μmol photons m^-2^ s^-1^ (*I*_15_), respectively.** **(A)** cell concentration (cells ml^-1^), **(B)** cellular Chl *a* concentration (pg Chl *a* cell^-1^), **(C)** total Chl *a* concentration (ng Chl *a* ml^-1^). Data points represent treatment means and error bars indicate standard errors (*n* = 3).

No ciliate prey cells were observed during enumeration of *Dinophysis* cells for the entire duration the prey starvation experiment. *D. acuta* cultures subjected to prey starvation at high photon irradiance (*I*_100_ = 100 μmol photons m^-2^ s^-1^) increased from 200 to 1400 cells ml^-1^ during 1 month of incubation, equivalent to an average growth of 2.8 cell divisions in total (Experiment 1, **Figure 1A**). The subculture that was exposed to low photon irradiance (*I*_15_ = 15 μmol photons m^-2^ s^-1^) increased in cell concentration from 200 to 1000 cells ml^-1^ and thus had <2.3 cell divisions during the 1 month of incubation (**Figure 1A**). The cellular Chl *a* concentration in *D. acuta* incubated at *I*_100_ decreased exponentially from the initial ∼53–16 pg Chl *a* cell^-1^ (**Figure 1B**), leading to an overall increase in Chl *a* concentration from 11 to a maximum of 26 ng Chl *a* ml^-1^ at Day 16 (**Figure 1C**). Cells incubated at *I*_15_, however, maintained their cellular Chl *a* concentration during the 1 month long incubation, even though cells divided 2.3 times. This led to an increase in the amount of Chl *a* in these incubations from 11 to 46 ng Chl *a* ml^-1^ at Day 30, indicating a higher net production of Chl *a* production in prey-starved *Dinophysis* cells grown at *I*_15_, compared to those at *I*_100_ (**Figure 1C**). For comparison, well fed cultures of *D. acuta* grown at *I*_100_ had a cellular Chl *a* concentration of ∼40 pg Chl *a* cell^-1^ (Experiment 2).

**FIGURE 2:**
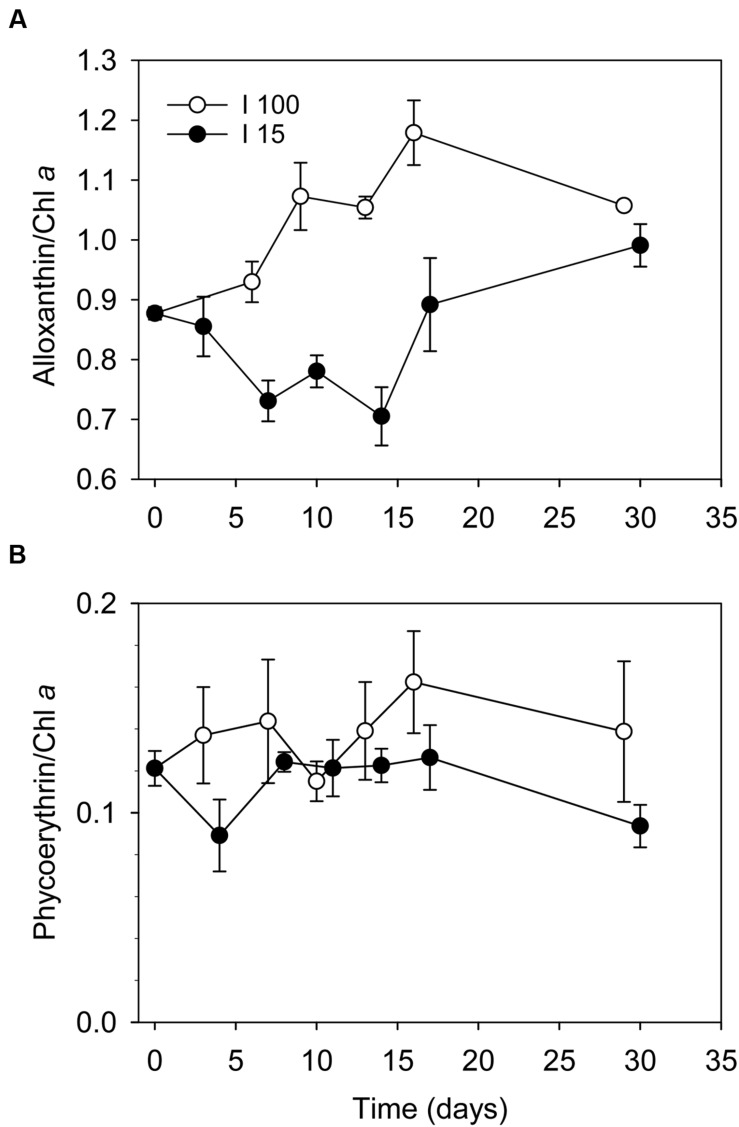
**Changes in the ratio of **(A)** alloxanthin:Chl *a* ratio, and **(B)** phycoerythrine:Chl *a* ratio in *D. acuta* cells during the starvation experiment under irradiances of *I*_100_**(o)** and *I*_15_**(●)**, respectively.** Data points represent treatment means, and error bars indicate standard errors (*n* = 3).

Cultures exposed to *I*_100_ exhibited a slight increase in the alloxanthin:Chl *a* ratio from 0.86 to ∼1.2 over the first 10 days. Hereafter, the ratio remained constant indicating that Chl *a* was initially lost at a faster rate than alloxanthin (**Figure 2A**). With cultures exposed to *I*_15_, a significant decrease in the alloxanthin:Chl *a* ratio from 0.86 to 0.7 was observed over the first 2 weeks indicating dilution due to growth, which was followed by an increase of the alloxanthin:Chl *a* ratio reaching ∼1 at the end of the experiment. *In vivo* measurements of Chl *a* and phycoerythrin showed that the changes in phycoerythrin content under the two experimental irradiance regimes matched those found in Chl *a* (**Figure 2B**).

### Single-Cell Variable Chlorophyll Fluorescence Imaging

**FIGURE 3:**
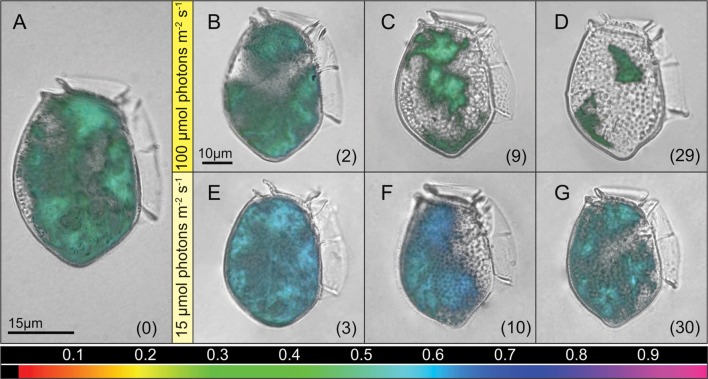
**Single cell variable chlorophyll fluorescence imaging.** Maximum PSII quantum yield (*F*_v_/*F*_m_) of single *D. acuta* cells during the starvation experiment. Images display cells at different time points with the quantum yield as an overlay in false color (values corresponding to the color scalebar). A culture representative image for fed cells growing at *I*_100_ prior to starvation **(A)**, and culture representative images for starved cells after transfer to irradiances of *I*_100_ **(B–D)** and *I*_15_ **(E–G)**. Numbers in brackets presents days of starvation, and time maintained under the given irradiance.

In well fed cultures of *D. acuta*, incubated at an irridiance of 100 μmol photons m^-2^ s^-1^, microscopy revealed a broad distribution of cellular Chl *a* with hotspots clearly defining 4–5 chloroplast centers with high fluorescence. Maximum PSII quantum yields (*F*_v_/*F*_m_) of ∼0.5 in these centers indicated a healthy and functioning photosynthetic apparatus (**Figure 3A**). During the starvation experiment at *I*_100_, we found a continuously declining Chl *a* coverage in the cells with incubation time (**Figures 3B–D**). After a slight increase in *F*_v_/*F*_m_ over the first 2 days, *F*_v_/*F*_m_ decreased with the declining chlorophyll coverage (**Figure 3B**; **Supplementary Figure S1**). At *I*_15_ we observed a rapid increase in *F*_v_/*F*_m_ to ∼0.6 over the first 3 days, which remained high even after 10 days, while the Chl *a* coverage had decreased a bit (**Figures 3E,F**; **Supplementary Figure S1**). After 30 days, we still observed an even coverage, almost similar to the well-fed cells, with similar condensation in four chloroplast centers, and with a sustained high level of *F*_v_/*F*_m_ (**Figure 3G**).

### Bulk Measurements of Photochemical and Non-photochemical Quenching

**FIGURE 4:**
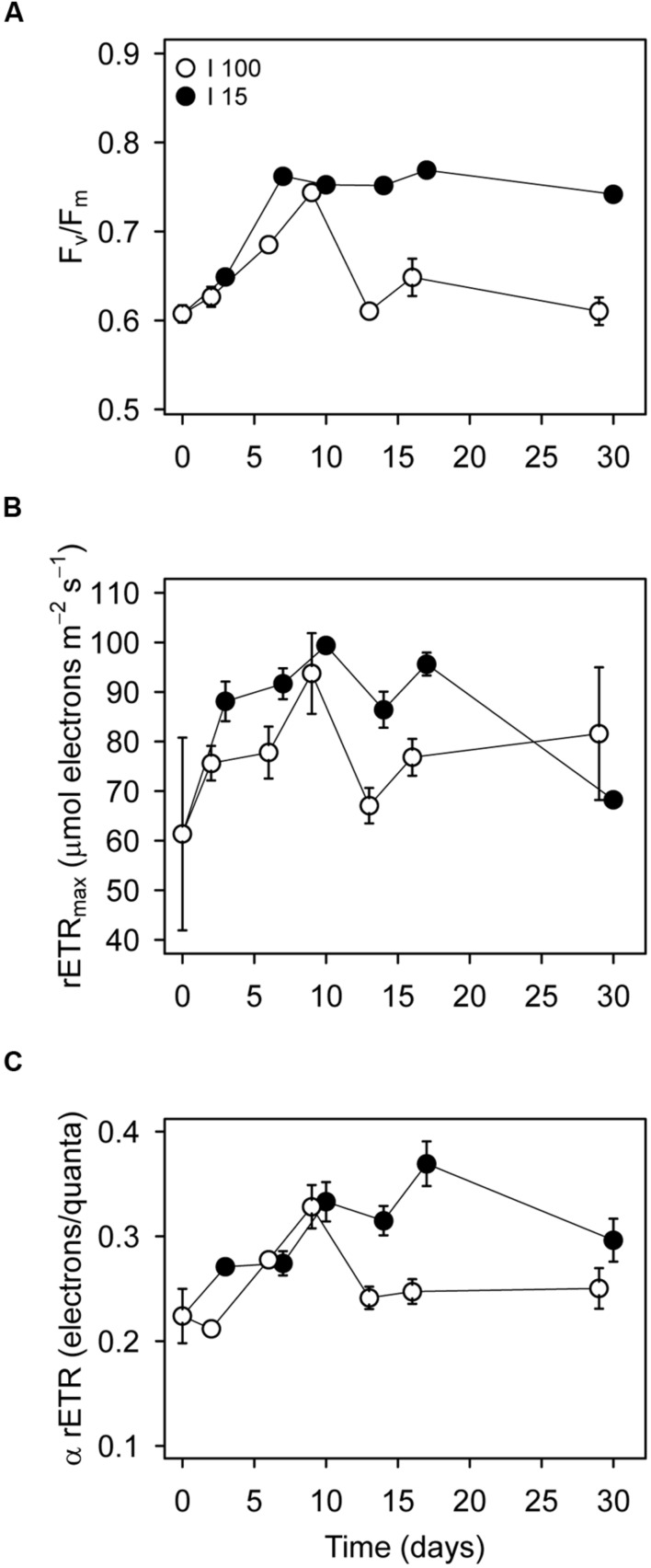
**Variable chlorophyll fluorescence measurements on *D. acuta* culture samples incubated at *I*_100_ (o) and *I*_15_**(●)** as a function of time after initiation of prey starvation.** The culture had been growing at *I*_100_ prior to the initiation of the experiment. **(A)** Maximum PSII quantum yield (*F*_v_/*F*_m_). **(B)** Maximum relative electron transport rates (rETRmax) and **(C)** initial slopes of the light response curves (α rETR), i.e., rETR vs irradiance. Data points represent the mean value parameter estimates obtained from the rapid light response curves. Error bars show standard error of the mean (*n* = 3).

Measurements on bulk cell samples yielded more insight into the photochemical, and non-photochemical quenching processes in starved *D. acuta* cultures. The maximum PSII quantum yield (*F*_v_/*F*_m_) was on average higher for cells at *I*_15_ (0.74 ± 0.04; average ± SE, *n* = 18) than *I*_100_ (0.65 ± 0.05, *n* = 21; **Figure 4A**). In both treatments, *F*_v_/*F*_m_ showed an initial increase during the 1 week, but only cells at *I*_15_ were able to maintain a high *F*_v_/*F*_m_ of ∼0.76, indicating better photosynthetic capacity than cells at *I*_100_. The change in *F*_v_/*F*_m_ resulted primarily from the change in the fraction of “open” PSII reaction centers, qP (**Supplementary Figure S2A**). An increase in qP to 0.94 ± 0.02 (*n* = 15) at *I*_15_ indicates a decrease in the proportion of “closed” PSII centers due to lower excitation pressure, than compared to cells at *I*_100_, which had a lower qP of 0.67 ± 0.02 (*n* = 21) throughout the starvation experiment.

Absorbed light energy partitioning profiles for *D. acuta* (**Supplement Figures S2A,B**) show a photoregulative change in response to changes in photon irradiance. The use of excitation energy for photosynthesis (ϕ_II_) in cultures kept at *I*_100_ was lowered with respect to *F*_v_/*F*_m_ by partial closure of PSII centers (**Supplementary Figure S2A**), and non-photochemical energy losses (ϕ_NPQ_ and ϕ_NO_) induced by illumination. Only 38% of the absorbed light energy was used for photosynthetic reactions (**Supplementary Figure S2B**), whereas cells kept at *I*_15_ used 70% of the absorbed light for photosynthesis (**Supplementary Figure S2C**).

The maximum relative electron transport rate (rETR_max_), and the initial slope of the light response curves (α rETR) showed no changes between the treatments (**Figures 4B,C**). For cultures incubated at *I*_100_, the average rETR_max_ and α rETR values were 76.3 ± 8.14 and 0.25 ± 0.01 (*n* = 21), respectively. Cultures at *I*_15_ showed average rETR_max_ and α rETR values of 88.2 ± 2.58 and 0.31 ± 0.02 (*n* = 18), respectively, (**Figures 4B,C**).

### Inorganic Carbon Uptake and Respiration before and during Starvation

**FIGURE 5:**
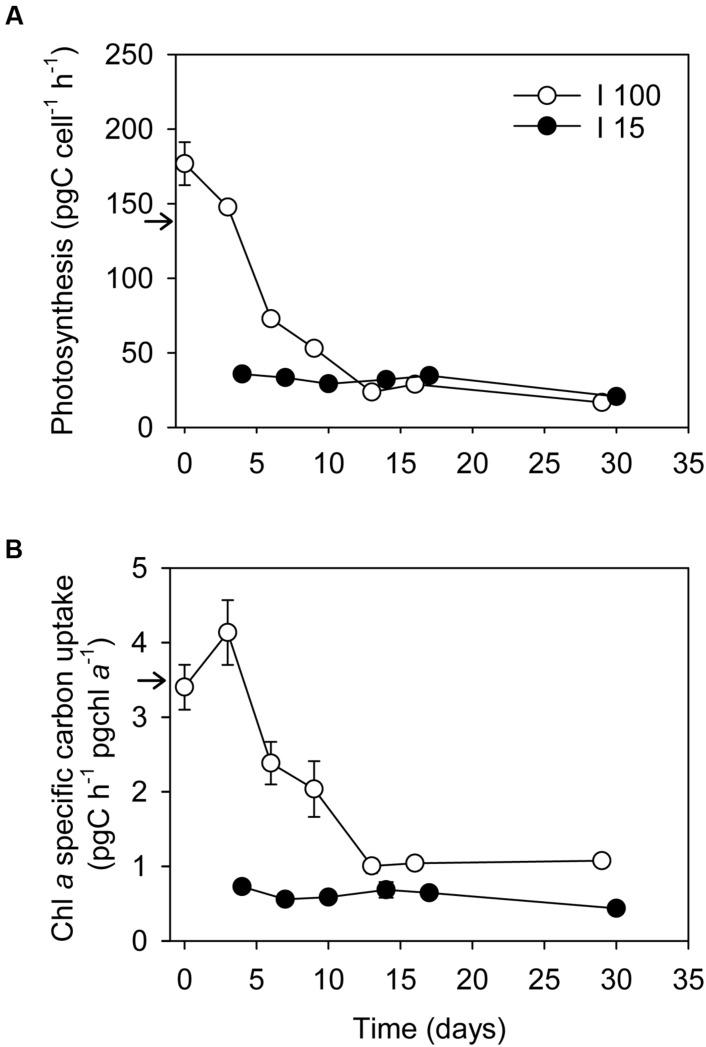
**Changes in **(A)** Cellular inorganic carbon uptake (pg C cell^-1^ h^-1^), and **(B)** Chl *a*-specific inorganic carbon uptake (pg C pg Chl *a*^-1^ h^-1^) in *D. acuta* during the prey starvation experiment at two irradiances, *I*_100_**(o)** and *I*_15_**(●)**, respectively.** Data points represent treatment means and error bars indicate standard errors (*n* = 3). Arrows indicate values from well fed cultures for comparison.

Inorganic carbon uptake was 138 ± 6 pg C cell^-1^ h^-1^ (average ± SE) in well fed cultures of *D. acuta* at *I*_100_, equivalent to 2.21 ± 0.94 ng C cell^-1^ day^-1^ (light:dark cycle of 16:8 h), leading to a Chl *a* specific rate of 3.5 ± 0.5 pg C pg Chl *a*^-1^. The inorganic carbon uptake of *D. acuta* cells subjected to starvation at *I*_100_ decreased exponentially from 177 ± 14 to 23.7 ± 4.2 pg C cell^-1^ h^-1^ at Day 16 (equivalent to 2.83 ± 0.23 and 0.38 ng C cell^-1^day^-1^, respectively, **Figure 5A**), i.e., a 6.1-fold decrease in photosynthetic carbon fixation. This translated into a significant decline in the Chl *a*-specific carbon uptake from the initial 3.4 to 1.0 pg C pg Chl *a*^-1^ h^-1^ at Day 16 (one-way ANOVA, *P* < 0.05; **Figure 5B**). The following 2 weeks, the inorganic carbon uptake rate dropped further to 16.8 ± 1.1 pg C cell^-1^ h^-1^ (=0.27 ± 0.02 ng C cell^-1^ day^-1^), but the Chl *a*-specific carbon uptake stayed constant (1.07 pg C pg Chl *a*^-1^ h^-1^).

Under low irradiance (*I*_15_), inorganic carbon uptake remained constant for the first 17 days at 33.1 ± 2.8 pg C cell^-1^ h^-1^ (=0.53 ± 0.03 ng C cell^-1^ day^-1^), and dropped only marginally to 20.7 ± 1.9 pg C cell^-1^ h^-1^ (=0.33 ± 0.03 ng C cell^-1^ day^-1^) after 1 month of incubation. Thus, the Chl *a-*specific inorganic carbon uptake rates of the starved low irradiance *D. acuta* stayed constant at ∼0.6–0.7 pg C μg Chl *a*^-1^ h^-1^ during the entire experiment.

Well-fed cells of *D. acuta* had a respiration rate of 86.5 ± 0.16 pg C cell^-1^ h^-1^ at *I*_100_, equivalent to 2.08 ± 0.38 ng C cell^-1^ day^-1^ (average ± SE, *n* = 9). No difference in respiration rate was observed as a function of prey starvation after 1 day of starvation in either of the light treatments, thus data were merged for each irradiance (One way-ANOVA, *P* > 0.05). Data on respiration of prey starved cells are only available until day 16 and 17 for the high and low irradiances, respectively. Starved cells had approximately halved their respiration rates [42.9 ± 7.1 (*n* = 11) and 36.2 ± 6.6 (*n* = 12) pg C cell^-1^ h^-1^ at *I*_100_ and *I*_15_, respectively]; respiration rates at the two irradiances were not statistically different (Two way-ANOVA, *P* > 0.05).

## Discussion

The present study documents for the first time that kleptochloroplasts (without prey nuclear material) taken up by *D. acuta* exhibit photoregulation, where photosynthetic pigments are produced to improve growth under both high and low irradiances. However, we saw no changes in photosynthesis vs. irradiance parameters like α rETR or max rETR during 1 month of starvation, similar to what is usually found in regular algae displaying photoacclimation (e.g., MacIntyre et al., 2001). Thus, we found photoregulation but not true photoacclimation in *D. acuta*.

Under low irradiance, photoregulation enabled *D. acuta* to maintain their cellular Chl *a* and phycoerythrin contents even though cells divided several times. We also found that the concentration of the alloxanthin increased relative to Chl *a* in cells exposed to high irradiance, while this pigment was initially reduced in cells incubated under low irradiance. While the role of alloxanthin as a light harvesting or photoprotective pigment in cryptophytes is not well established, the few available reports suggest that the alloxanthin:Chl *a* ratio in cryptophytes is elevated at high irradiances indicating that it may have photoprotective properties (Schlüter et al., 2000; Laviale and Neveux, 2011) and alloxanthin may thus play a similar role in *D. acuta*.

The literature provides no evidence for division of kleptochloroplasts in *Dinophysis* spp., suggesting that the cellular number of chloroplasts will decrease during cell division (Minnhagen et al., 2008). Hence, the ability of *Dinophysis* to maintain cellular photosynthetic pigment concentrations may potentially lead to enlargement (rather than division) of individual chloroplasts (Nielsen et al., 2013). Enlargement of kleptochloroplasts has recently been shown in other dinoflagellates such as *Nusuttodinium aeruginosum* and *N. myriopyrenoides*, where a 20-fold enlargement of the chloroplasts was observed within 120 min after ingestion in *N. aeruginosum* (Yamaguchi et al., 2011; Onuma and Horiguchi, 2015). Up to 10-fold enlargement of ingested chloroplasts has also been observed in the katablepharid *Hatena arenicola* (Yamaguchi et al., 2011). However, in all these cases, prey nuclei and nucleomorphs were retained by the host cell, which is not the case in *Dinophysis* spp.

Variable chlorophyll fluorescence data suggested that chloroplasts in *D. acuta* were competent with maximum PSII quantum yields (*F*_v_/*F*_m_) between 0.5 and 0.8 throughout the duration of the experiment (30 days). These values are similar to what is usually found in well-functioning microalgae (Falkowski and Raven, 2007). Similar observations have been obtained on *D. caudata* cells starved for up to 85 days (Park et al., 2008), where *F*_v_/*F*_m_ began to decline after 24 days of starvation, and reached 0.4 after 45 days of starvation, i.e., at the same time as cell divisions stopped. Thereafter, *F*_v_/*F*_m_ decreased steadily over the next days and reached zero after 85 days of starvation. We saw no indications of photodamage. Photodamage would be characterized by a decrease in ϕ_NPQ_, which is one of the most important safety valves for the regulation of light harvest (Müller et al., 2001). Also, if the cells were photodamaged we would have expected to see a consecutive decrease of qP (Mackey et al., 2008), which gives information if photosynthetic efficiency has been altered by a changed proportion of functional reaction centers. In our case, ϕ_NPQ_ was relatively stable (∼20%) and qP was constant in high light (60–70%) throughout the experiment, so the cells were unlikely to experience photodamage.

The prey species of *Dinophysis* spp., i.e., the red *Mesodinium* spp. retain ingested cryptophyte nuclei for up to 100 days and these have been shown to be transcriptionally active (Johnson et al., 2007; Lasek-Nesselquist et al., 2015). However, it has also been shown that the expression of a cryptophyte nuclear-encoded gene for the plastid-targeted protein, *LHCC10*, involved in plastid function, declined in *Mesodinium* cells as the sequestered nuclei disappeared from the population (Johnson et al., 2007). In the red *Mesodinium* spp., *F*_v_/*F*_m_ values of 0.54–0.66 have been observed when grown for up to a month without prey (Johnson et al., 2006; Kim et al., 2014).

Another kleptochloroplastidic dinoflagellate, *Amylax triacantha*, also preys on red *Mesodinium* spp. like *Dinophysis* spp. (Kim et al., 2014). Besides the cryptophyte chloroplasts, *A. triacantha* also retains cryptophyte mitochondria, cryptophyte nuclei and nucleomorphs. However, unlike in *M. rubrum*, the ingested cryptophytes are kept as individual packages. In *A. triacantha* cultures subjected to prey starvation, *F*_v_/*F*_m_ remains at 0.6 for the first 5–8 days, whereafter it declines over time to reach *F*_v_/*F*_m_ values of ∼0.05 after 25 days of prey starvation in conjunction with the loss of cryptophyte nuclear material, i.e., a much faster decline in photosynthetic capacity compared to the red *Mesodinium* spp.

Available evidence thus suggests that the cryptophyte chloroplasts are not independent entities, which will function on their own inside a host cell. In the red *Mesodinium* spp. and in *A. triacantha* the photosynthetic performance of kleptochloroplasts is coupled to the sequestration of prey nuclei and nucleomorph material. This is not the case in *Dinophysis* spp. Thus, it seems that the function of chloroplasts in *Dinophysis* may depend upon genes, which in the past have been transferred from prey nuclei and nucleomorphs to the dinoflagellate genome.

Very little is known about the transfer of genes from the cryptophyte genome to the genome of *Dinophysis* spp., and only one species, *D. acuminata*, has been investigated (Wisecaver and Hackett, 2010). Five proteins, complete with plastid-targeting peptides that may function in photosystem stabilization and metabolite transport, have been found encoded in the nuclear genome of *D. acuminata* (Wisecaver and Hackett, 2010). It seems unlikely that those genes alone allow for the extensive regulation of photosynthetic and photoprotective pigments that we observed in *D. acuta*, and more genes controlling photoregulation in *D. acuta* and other *Dinophysis* spp. remain to be identified.

The Chl *a*-specific inorganic carbon uptake dropped dramatically in cells grown at high irradiance indicating that, although *D. acuta* was able to produce Chl *a*, this did not translate into increased inorganic carbon uptake. There may be several reasons for this observation: First, since *Dinophysis* spp. only sequester the chloroplasts from their prey, any loss of genes involved in the regulation of RuBisCO that are located in the cryptophyte nuclei material are lost and this will strongly affect inorganic carbon fixation. Such genes may come from the prey nucleus, but they may also come from the nucleomorph. The Rubisco regulatory protein, CbbX, for instance, is encoded by the cryptophyte nucleomorph in cryptophytes (Maier et al., 2000).

Second, RuBisCo is located in the single terminally placed pyrenoid, contained by every chloroplast in most algae (Holdsworth, 1971; Giordano et al., 2005; Garcia-Cuetos et al., 2010). As *D. acuta* cannot divide the aquired chloroplasts, it will also be unable to divide its pyrenoids, which seems to be a key missing control factor, lacked by these protists to fully regulate and divide the sequestered “third hand” chloroplasts.

In purely phototrophic dinoflagellates, respiration rates typically account for ∼20 and ∼40% of gross photosynthesis in the exponential and stationary growth phase, respectively (Geider and Osborne, 1989; Geider, 1992; López-Sandoval et al., 2014). In the kleptochloroplastidic *D. acuta*, respiration rates accounted for ∼50% of gross photosynthesis in well fed cells, and that percentage increased when the cells were subjected to prey starvation. Under high irradiance, *D. acuta* cells became full of storage material (unpublished observations, probably lipids and starch; Durand-Clement et al., 1988) upon initiation of prey starvation, in accordance with earlier observations in *D. caudata* (Park et al., 2008). Accordingly, we found excess inorganic carbon uptake during the first ∼9 days under high irradiance, whereafter respiration rates started to slightly exceed rates of inorganic carbon uptake. Under low irradiance, respiration rates slightly exceeded the inorganic carbon uptake at all times. The build up of carbon storage upon onset of prey starvation and maintenance of fully active kleptochloroplasts thus allows *D. acuta* to survive for extended periods of time (months; Park et al., 2008; Nielsen et al., 2012, 2013). This is an important trait of these kleptochloroplastidic dinoflagellates, and explains how they can survive in a fluctuating environment and still depend upon *M. rubrum* as a single type of prey.

## Conclusion

We found strong evidence of photoregulation via production of photosynthetic and photoprotective pigments in the klepto-chloroplastidic dinoflagellate, *D. acuta*, which only retains the chloroplasts from its prey. No direct evidence of changes in photosynthetis vs. irradiance parameters and thus no signs of photoacclimation were observed in *D. acuta* incubated at different irradiances, and thus no signs of photoacclimation were found. A decrease in Chl *a*-specific inorganic carbon uptake in prey-starved cells indicated a dilution of carbon fixing units (Rubisco) among daughter cells in combination with the production of Chl *a*. However, in their natural environment *D. acuta* and the >100 species that belong to the genus *Dinophysis* may also indirectly photoregulate via increased retention of kleptochloroplasts, when prey cells are available. It is also possible that *Dinophysis* cells may indirectly achieve “photoacclimation” by ingestion of photoacclimated prey cells and maintaining a higher number of chloroplasts. Our data point to a hitherto unstudied role of gene transfer from prey to *D. acuta* that may enable it to regulate the function of its kleptochloroplast, although the exact genetic and biochemical mechanisms remain to be identified.

## Author Contributions

All authors were involved in the design and planning of the experiments. PH prepared the cultures for experimentation and was in charge of sampling scheme, carried out all ^14^C measurements and data analysis, except for the last sampling where TB carried out the measurements. TB did all respiration measurements and data analysis, isolation of cells for all pigment analyses and carried out fluorometric measurements and connected data analysis of phytopigments. ET carried out all Imaging PAM and HPLC measurements of phytopigments and the connected data analysis. KO carried out all MultiColorPAM measurements and the connected data analysis. PH wrote the manuscript with inputs from all of the co-authors.

## Conflict of Interest Statement

The authors declare that the research was conducted in the absence of any commercial or financial relationships that could be construed as a potential conflict of interest.
