# Supplementary material for: Photoregulation in a Kleptochloroplastidic Dinoflagellate, Dinophysis acuta
Source: Front Microbiol. 2016 May 30;7:785. doi: 10.3389/fmicb.2016.00785 (PMC4884750; doi:10.3389/fmicb.2016.00785)
Supplement: Supplementary file 1 [file Data_Sheet_1.DOCX]

***Supplementary Material***

**Photoregulation in a Kleptochloroplastidic Dinoflagellate, *Dinophysis acuta***

**Per J. Hansen^1,^*, Karin Ojamäe^2^, ^1^Terje Berge^1,3^, Erik C.L. Trampe^1^, Lasse T. Nielsen^3^, Inga Lips^2^, Michael Kühl^1,4^**

*** Correspondence:** Per J. Hansen: pjhansen@bio.ku.dk

1. **Supplementary Text**

**1.1 Material and methods**

**1.1.1 Cuvette-based variable chlorophyll fluorescence measurements**

Standard variable chlorophyll fluorescence measurements and determination of relative PSII electron transport rates were done with a MULTI-COLOR-PAM chlorophyll fluorometer (Heinz Walz GmbH, Effeltrich, Germany) following the saturation pulse method (Schreiber et al., 1986).

The maximum quantum yield of PSII (F_v_/F_m_) was obtained from measurements on 20 min dark-adapted cells. For measurements of the minimum chlorophyll fluorescence yield (F_0_), samples were pre-illuminated with weak far-red light (725 nm; 5 sec, 1.0 μmol photons m^-2^ s^-1^) that excites Photosystem I (PSI) and thus oxidizes the plastoquinone and quinone A (Q_A_) pools associated with PSII. After the brief far-red light illumination, a saturating blue light pulse (440 nm; 300 ms, 5710 μmol photons m^-2^ s^-1^) was instantly delivered, closing all PSII reaction centers for measurement of the 15 maximum chlorophyll fluorescence yield (F_m_). F_0_ and F_m_ were measured three times for each experiment flask with 30 sec intervals allowing reoxidation of the PSII reaction centers between saturation pulses. F_v_/F_m_ was calculated according to Kitajima and Butler (1975):

$\frac{F_{v}}{F_{m}}=\frac{F_{m}-F_{0}}{F_{m}}$ (S1)

Equation (S1) describes the potential photochemical efficiency of open PSII reaction centers. The effective photochemical quantum yield of PSII, Y(II), was obtained from light adapted samples where the steady-state chlorophyll fluorescence yield (F) was measured after exposing samples to actinic light (440 nm; 115 and 18 μmol photons m^-2^ s^-1^ for high and low light cultures respectively) for 30 sec. This illumination time was long enough to allow the fluorescence yield to stabilize at a constant level, F. Subsequently, a saturating light pulse was applied and the maximum chlorophyll fluorescence yield in the light-adapted state was determined (F_m_’). Subsequently, φ(II) was calculated according to Genty et al. (1989):

$\varphi(II)=\frac{F_{m}'-F}{F_{m}'}$ (S2)
Equation (S2) describes the proportion of absorbed light energy being used in photochemistry, i.e., it is a measure of the operating efficiency of PSII once steady-state electron transport has been achieved at any given irradiance. The quantum yield of non-regulated energy quenching was obtained according to Hendrickson et al. (2004):

$\varphi(NO)=\frac{F}{F_{m}}$ (S3)
Here, φNO quantified the thermal energy dissipation that occurs even when potential photochemical yield is maximal. The quantum yield of light-induced non-photochemical quenching was calculated according to Hendrickson et al. (2004):

$\varphi(NPQ)=\frac{(F/F_{m}')}{(F/F_{m})}$ (S4)

where φNPQ is defined as the regulated energy losses of excitation energy via heat dissipation involving ∆pH- and xanthophyll-like energy dissipating mechanisms. The sum of all yields for the dissipative processes acting on the energy absorbed by PSII is unity:

$\varphi(II)+\varphi(NO)+\varphi(NPQ)=1$ (S5)

Photochemical chlorophyll fluorescence quenching (qP) was obtained according to Schreiber et al. (1986):

$qP=\frac{F_{m}'-F}{F_{m}'-F_{0}'}$ (S6)

This parameter estimates the fraction of oxidized PSII reaction centers. The minimum chlorophyll fluorescence yield of illuminated sample (F_0_’) was estimated according to Oxborough and Baker (1997):

$F_{0}'=\frac{1}{(1/F_{0}-1/F_{m}+1/F_{m}')}$ (S7)

For relative electron transport rate (rETR) measurements, samples were sequentially exposed to 14 irradiance levels (E) from 0 to 512 μmol m^-2^ s^-1^ for 30 sec at each step. rETR and the effective photochemical quantum yield was calculated for each irradiance. The relative electron transport rate is a measure of the relative rate of linear electron flow through PSII, without any information on absolute rate. Originally, rETR as a fluorescence-derived parameter was introduced for PAM- measurements with leaves (Schreiber et al., 1994):

$rETR=\varphi(II)\times E$ (S8)

The obtained light response curves, i.e. rETR vs irradiance curves were fitted using the model of Eilers and Peeters (1988) in order to derive fitting parameters for the initial slope (α), inhibition term (β), light saturation parameter (E_k_) and for the maximum relative electron transport rate (rETR_max_). All parameters were measured after subtraction of the blank fluorescence, measured on sterile-filtered f/2 medium.

**1.1.2 Microscopic variable chlorophyll fluorescence imaging**

The photosynthetic capacity of single *D. acuta* cells was monitored by variable chlorophyll fluorescence imaging using a multicolor pulse-amplitude modulated imaging system (RGB Microscopy I-PAM, Heinz Walz GmbH, Effeltrich, Germany) mounted on an epifluorescence microscope (Axiostar Plus FL, Carl Zeiss GmbH, Germany) fitted with a 100x/1.30 Oil Ph3 UPlanFL objective (Olympus Europa Holding GmbH, Hamburg, Germany). A detailed description of the system is presented in Trampe et al. (2015). Actinic, saturating and measuring light pulses were provided by a red-green-blue LED lamp (IMAG-RGB; Heinz Walz GmbH, Effeltrich, Germany) attached to the epifluorescence port of the microscope. The camera and LED lamp were connected to a control unit (IMAG-CM; Heinz Walz GmbH, Effeltrich, Germany) controlled by dedicated PC software (Imaging-WIN 2.41; Heinz Walz GmbH, Effeltrich, Germany). To keep a constant temperature of 12°C throughout measurements, we fitted a custom-made thermostatic slide holder to the microscope cross-table (see Trampe et al., 2011). All variable chlorophyll fluorescence imaging measurements in our experiment were conducted with the red, green and blue LED’s running in concert, i.e. white light, to increase the relatively low fluorescence signal at this scale. Chlorophyll fluorescence imaging systems have been described in detail elsewhere (Schreiber, 1994: Kühl and Polerecky, 2008, Trampe et al., 2011).

*Measurements.* One to four *D. acuta* cells were located on the microscope using the bright field halogen light source of the microscope covered with a 715 nm long-pass glass filter (RG715, Schott GmbH, Germany) to avoid actinic light exposure during focusing, and were then dark-adapted for 15 min prior to all measurements. This allowed for all reaction centers of PSII to open, and we first imaged the minimal fluorescence yield (F_0_). A subsequent saturation pulse closed all reaction centres, and we could image the maximal fluorescence yield (F_m_). These images then allowed us to generate images of the maximal PSII quantum yield, F_v_/F_m_, showing in false colour coding. These images where exported, and combined with corresponding monochromatic images as overlays to produce a detailed overview of internal chloroplast location in single *D. acuta* cells. Areas of interest (AOI) were defined to cover the photosynthetic active part of a single cell enabling averaging of F_v_/F_m_ values over the AOI.

**1.2 Results**

**1.2.1 Photosynthetic capacity of single cells**

The development of the photosynthetic capacity in *D. acuta* cultures during starvation is shown in Supplementary figure S1, as derived from variable chlorophyll fluorescence imaging of F_v_/F_m_ in single cells. Averages of 26-38 single cells were calculated from defined AOI’s at each time point. The photosynthetic capacity measured as F_v_/F_m_ increased significantly at the beginning of the starvation experiment when cultures were placed under two different light regimes. The cells growing at I_15_ displayed the strongest initial increase due to a rapid up-regulation of the photosynthetic apparatus, optimizing for the relatively low light. Subsequently, the maximum quantum yield reaches a constant level before decreasing a bit and showing a higher intercellular variation towards the end of the experiment after 30 days. There was a similar but more modest increase in photosynthetic capacity for cells growing at I_100_. The curve indicates less optimal conditions for the starving cells at this higher irradiance level, and F_v_/F_m_ exhibited a stronger and faster decrease than in starved *D. acuta* exposed to lower light, stabilizing toward the end of the experiment at a significantly lower level.

**2. Supplementary Figures**

**Supplementary Figure 1.** Maximum PSII quantum yield of *D. acuta* cells during the starvation experiment, quantified from microscopic variable fluorescence imaging of individual *D. acuta* cells grown in different light environments: I_15_ (filled symbols), and I_100_ (open symbols). Data points represent treatment means and error bars refer to standard error of the mean (*n*=26-38).

**Supplementary Figure 2.** Variable chlorophyll fluorescence measurements of *D. acuta* incubated during starvation experiment, quantified from cuvette-based variable fluorescence measurements at I_15_ (filled symbols) and I_100_ (open symbols) as a function of time. Photochemical quenching of fluorescence (qP) for starved *D. acuta* cells (A), partitioning of absorbed light energy of PSII into quantum yields of photochemistry (φII), non-regulated (φNO) and regulated (φNPQ) non-photochemical quenching at I_100_ (B) and I_15_ (C). Data points represent means and error bars refer to standard error of the mean (*n*=3).

**3. References not cited in the main article**

Eilers, P.H.C., Peeters, J.C.H. (1988). A model for the relationship between light intensity and the rate of photosynthesis in phytoplankton. *Ecol. Model.* **42**, 199-215.

Genty, B., Briantais, J.M., Baker, N.R. (1989). The relationship between the quantum yield of photosynthetic electron transport and of quenching chlorophyll fluorescence. *Biochim. Biophys. Acta* **990**, 87–92.

Hendrickson, L., Furbank, R.T., and Chow, W. S. (2004). A simple alternative approach to assessing the fate of absorbed light energy using chlorophyll fluorescence. *Photosynth. Res*. **82**, 73-81.

Kitajima, M., Butler, W.L. (1975). Quenching of chlorophyll fluorescence and primary photochemistry in chloroplasts by dibromothymoquinone. *Biochimica et Biophysica Acta* **376**, 105-115.

Kühl, M., Polerecky, L. (2008). Functional and structural imaging of phototrophic microbial communities and symbioses. *Aquat. Microb. Ecol.* **53**, 99-118.

Oxborough, K., Baker, N.R. (1997). Resolving chlorophyll a fluorescence images of photosynthetic efficiency into photochemical and non-photochemical components – calculation of qP and Fv’/Fm’ without measuring F0’. *Photosynt. Res.* **54**, 135-142.

Schreiber, U., Schliwa, U., Bilger, W. (1986). Continuous recording of photochemical and non-photochemical chlorophyll fluorescence quenching with a new type of modulation fluorometer. *Photosynt. Res.* **10**, 51-62.
